# Higher resource level promotes virulence in an environmentally transmitted bacterial fish pathogen

**DOI:** 10.1111/eva.12466

**Published:** 2017-03-30

**Authors:** Hanna Kinnula, Johanna Mappes, Janne K. Valkonen, Katja Pulkkinen, Lotta‐Riina Sundberg

**Affiliations:** ^1^ Department of Biological and Environmental ScienceCentre of Excellence in Biological InteractionsUniversity of JyvaskylaJyvaskylaFinland

**Keywords:** aquaculture, bacteria, environment, infection, nutrient, virulence

## Abstract

Diseases have become a primary constraint to sustainable aquaculture, but remarkably little attention has been paid to a broad class of pathogens: the opportunists. Opportunists often persist in the environment outside the host, and their pathogenic features are influenced by changes in the environment. To test how environmental nutrient levels influence virulence, we used strains of *Flavobacterium columnare*, an environmentally transmitted fish pathogen, to infect rainbow trout and zebra fish in two different nutrient concentrations. To separate the effects of dose and nutrients, we used three infective doses and studied the growth of bacteria in vitro. High nutrient concentration promoted both the virulence and the outside‐host growth of the pathogen, most notably in a low‐virulence strain. The increase in virulence could not be exhaustively explained by the increased dose under higher nutrient supply, suggesting virulence factor activation. In aquaculture settings, accumulation of organic material in rearing units can locally increase water nutrient concentration and therefore increase disease risk as a response to elevated bacterial density and virulence factor activation. Our results highlight the role of increased nutrients in outside‐host environment as a selective agent for higher virulence and faster evolutionary rate in opportunistic pathogens.

## 1 Introduction

Ecological changes arising from modern agricultural and intensive farming practices are among the most commonly identified factors in disease emergence, due to dynamic interactions between rapidly adapting microorganisms and changes in the environment (Kennedy et al., 2016; Le Bouguenec & Schouler, 2011; Mennerat, Nilsen, Ebert, & Skorping, 2010; Morens, Folkers, & Fauci, 2004; Morse, 1995; Schrag & Wiener, 1995). One example of such an ecological change is the anthropogenic nutrient enrichment of aquatic environments (Johnson et al., 2010) that has been shown to increase the risk of infection or disease severity (Bruno, Petes, Harvell, & Hettinger, 2003; Johnson et al., 2007; McKenzie & Townsend, 2007; Voss & Richardson, 2006; Wedekind, Gessner, Vazquez, Maerki, & Steiner, 2010). The effects of increased nutrient availability on disease are diverse and depend on several factors, such as the types of pathogen and host, host condition, characteristics of the aquatic system, and the degree of nutrient enrichment (Johnson & Carpenter, 2010; Johnson et al., 2010). Generalist or opportunistic pathogens that are not dependent on a single host have especially been suggested to benefit from increased nutrient availability (Johnson & Carpenter, 2010; Johnson et al., 2010). These kinds of pathogens are particularly harmful, as they may cause continuous outbreaks or declines in host populations without reductions in pathogen transmission (Brown, Cornforth, & Mideo, 2012; Johnson et al., 2010). Although the effects of increased nutrient supply on disease are commonly well acknowledged, the interactions between nutrients and environmental bacteria, and the evolutionary mechanisms through which increased nutrients influence the emergence of disease in aquatic systems have remained poorly understood (Johnson et al., 2010; Llafferty & Holt, 2003; McKenzie & Townsend, 2007; Smith & Schindler, 2009; Smith, Tyrees, & Smith, 2005).

Increased nutrient supply can directly influence replication, survival, population structure, and virulence of the opportunistic pathogens that are able to shuttle between the outside‐host and within‐host environments (Brown et al., 2012; Smith & Schindler, 2009). The opportunists are often also able to respond to altered conditions by changing their phenotypic expression, such as virulence. Several observational and experimental studies have shown that nutrient inputs in aquatic environments may increase bacterial biomass (see e.g., Elser, Stabler, & Hassett, 1995; Forehead, Kendrick, & Thompson, 2012; Li, Head, & Harrison, 2004; Sipura et al., 2005) or disease severity (Bruno et al., 2003; Johnson et al., 2007; Wedekind et al., 2010). However, the quality of resources in the outside‐host environment can also have a significant impact on the virulence of an opportunist pathogen (Ketola, Mikonranta, Laakso, & Mappes, 2016). Thus, nutrient availability may cause various responses in the virulence of different pathogen species and alter the risk of disease even at a local scale. In addition, outside‐host nutrients may accelerate the evolutionary rate of opportunists, compared to pathogens restricted to within‐host replication. Therefore, it is of particular importance to understand how environmental nutrient levels shape the dynamics and virulence of environmental opportunistic pathogens.

Aquaculture is the fastest growing food production system in the world, and diseases are the most significant constraint to its development. In fish farming, excess feeding to maximize production leads to accumulation of organic nutrients, such as nitrogen and phosphorus, in the water. Such a nutrient‐rich environment combined with intensive farming practices (e.g., high host densities) can promote virulence evolution in the fish‐pathogenic microbes and parasites (Nowak, 2007; Mennerat et al., 2010; Kennedy et al., 2016). *Flavobacterium columnare* (Bacteroidetes, Bernardet, and Grimont 1989) is the causative agent of columnaris disease in farmed freshwater fish worldwide (Declercq, Haesebrouck, Van den Broeck, Bossier, & Decostere, 2013; Pulkkinen et al., 2010; Wakabayashi, 1991). The disease transmits via water and biofilms from the environment and diseased fish (Cai, De La Fuente, & Arias, 2013; Kunttu, Suomalainen, Pulkkinen, & Valtonen, 2012). Therefore, environmental conditions are likely to affect *F. columnare* outbreaks and disease virulence (Wakabayashi, 1991), which can have mounting effects on the onset and persistence of disease epidemics in aquaculture. Yet, it is poorly understood how variation in the initial virulence of bacterial strains affects later responses to environmental nutrients.

Our study focuses on the influence of nutrients on infective dose and virulence, and on comparing the replication rate of opportunistic pathogen strains of low and high virulence in different nutritional environments. Special attention is paid on teasing apart the effects of dose and nutrients on pathogen virulence. This study provides empirical evidence on environmental factors that contribute to disease severity in a globally significant fish pathogen affecting aquaculture.

## 2 Materials and Methods

### 2.1 Bacteria and fish

Three previously isolated *F. columnare* strains were used in this study: a high‐virulence strain (B067) isolated from a diseased fish (Laanto, Sundberg, & Bamford, 2011), a high‐virulence strain (B185) isolated from tank water during a columnaris outbreak at a fish farm (Laanto et al., 2011), and a low‐virulence strain (B398) isolated from the inlet water of a fish farm (Kunttu et al., 2012). Pure cultures of the strains were stored frozen at −80°C in a stock containing 10% of glycerol and 10% of fetal calf serum. Before the experiments, the bacterial strains were revived overnight, after which the culture was inoculated 1:10 in a modified Shieh broth (Song, Fryer, & Rohovec, 1988; see Table S1) and grown at 26.0°C with constant agitation (150 rpm) for 21 hr.

Virulence experiments were performed using two fish host species, zebra fish (*Danio rerio*, Hamilton 1822) and rainbow trout (*Oncorhynchus mykiss*, Walbaum 1792). Adult, unsexed, disease‐free zebra fish (average weight 0.27 g ± 0.094) were obtained from the Core Facilities and Research Services of the University of Tampere 4 weeks before the experiment and maintained in 250‐L aquaria in aerated groundwater at a constant temperature of 25.0°C. Apparently healthy fingerling rainbow trout (average weight 3.83 g ± 1.562) were obtained from a fish farm in Central Finland during cold‐water season in early April when *F. columnare* does not cause epidemics in Finland. The fish had been maintained in *F. columnare*‐free groundwater at 15.0–17.5°C for 6 months prior to the experiments, during which time the fish showed no clinical signs of disease. To acclimatize the rainbow trout to the experimental conditions, the water temperature was gradually increased to 25.0°C over 3 days prior to the experiment (i.e., two and half degrees per day).

### 2.2 Virulence in fish hosts

The fish were challenged using a previously optimized continuous infection method, in which the overnight‐grown bacteria were directly added into each aquarium (see Laanto, Bamford, Ravantti, & Sundberg, 2015). Zebra fish were exposed to high‐virulence strains B067 and B185, and to the low‐virulence strain B398, and rainbow trout to strains B067 and B398. The fish were challenged and maintained one fish per aquarium in 0.5 L (zebra fish) or 1.0 L (rainbow trout) of aerated groundwater. Each bacterial strain was given to fish in doses of 1.0 × 10^4^, 1.0 × 10^5^, and 1.0 × 10^6^ colony‐forming units per milliliter (CFU ml^−1^), with two different growth medium additions to control for nutrient concentration. The volume of Shieh broth in the infections was either 6.4 ml/L (0.64%; low nutrient level) or 12.8 ml/L (1.28%; high nutrient level). Each strain*dose*resource treatment was performed in five replicates for zebra fish and in three replicates for rainbow trout. Thus, the experimental setup consisted of 18 treatment groups for zebra fish and 12 treatment groups for rainbow trout, totaling to 126 fish, of which 90 were zebra fish and 36 rainbow trout. After infection, the aquaria were randomly placed on shelves in the experimental room, and the rainbow trout aquaria were equipped with air pumps. To calculate the bacterial concentrations used in infections, the optical densities (OD) of the original overnight cultures were determined with a spectrophotometer (VICTOR X Multilabel Plate Reader, Perkin‐Elmer, USA) at wavelength of 570 nm. OD values were converted into CFU ml^−1^ using a previously determined relationship between OD and CFU (based on our unpublished experiments).

We monitored the fish for disease symptoms and morbidity for 4.5 days. This was performed at 2‐hr intervals during the first 48 hr. When the disease occurrence slowed down, the monitoring interval was extended accordingly, but the fish were checked at least four times per day. The water temperature was maintained between 25.0 and 26.0°C during the experiment. The moribund fish that did not react to external stimuli were put down by decapitation under terminal anesthesia with MS‐222 (Sigma). Also the fish surviving until the end of the experiment were euthanized. To verify *F. columnare* infection from the diseased fish, bacterial cultivations from fish gills were spread on Shieh agar plates supplemented with tobramycin (Decostere, Haesebrouck, & Devriese, 1997). The yellow colonies with the rhizoid morphology typical to *F. columnare* were considered as a confirmation of columnaris infection.

The experimental fish challenge was conducted according to the Finnish Act on the Use of Animals for Experimental Purposes, under permission ESAVI‐2010‐05569/Ym‐23, granted to L‐RS by the National Animal Experiment Board at the Regional State Administrative Agency for Southern Finland.

### 2.3 Bacterial replication in water

The effect of nutrient level on the replication of high‐virulence strain B067 and low‐virulence strain B398 was examined in vitro in three concentrations of modified Shieh broth. Before the experiment, the bacteria were grown for 21 hr in Shieh broth at 26°C with constant agitation (150 rpm). The study setting included three treatment groups. First, a volume of 68 μl of bacterial culture was added to 20 ml of autoclaved, dechlorinated tap water in sterile 50‐ml tubes to reach an initial bacterial count of 1 × 10^6^ CFU ml^−1^. This treatment served as the reference level without added nutrients (baseline level), having 0.34% of Shieh broth concentration originating from the 68 μl inoculation. The two other nutrient levels were equivalent to the concentrations used in the virulence experiment (low nutrient level with 0.64% and high nutrient level with 1.28% of growth medium). To reach these levels, 60 or 188 μl of Shieh broth was added into the reference‐level bacterial culture (totaling 128 or 256 μl, respectively). All the treatments were performed in triplicate. The initial bacterial count was determined with a spectrophotometer in a similar way as in the virulence experiment and confirmed by plate counting. Tap water was used in this experiment, as the bacterial strains failed to survive in sterilized distilled water.

The cultures were incubated for 5 days at 25.0°C under constant, gentle shaking (50 rpm). To examine the influence of nutrient level on bacterial replication, a sample of 100 μl was taken from each culture after 24, 48, 72, 96, and 120 hr, diluted to 10^−1^, 10^−2^, 10^−3^, 10^−4^, and 10^−5^, and spread on Shieh agar plates. The plates were incubated at 25.0°C for 48 hr, after which the bacterial colonies were counted manually.

### 2.4 Data analysis

To examine the effects of nutrient level and infection dose on host morbidity in the virulence experiment, we used a generalized linear model for binomial distribution. The morbidity risk of host within hour was modeled as a function of nutrient level (high or low), bacterial strain (high‐virulence strains B067 and B185, and low‐virulence strain B398), host species (rainbow trout, zebra fish), and infection dose (included as continuous covariate). To find the best model to explain the fate of fish, all the explanatory variables and all their interactions were first included in the model. The model was then simplified based on Akaike information criteria using a backward stepwise procedure (Table 1). To ensure that the observed morbidity was caused by *F. columnare* infection, the gill sample cultivations were analyzed by Pearson's chi‐squared test with Yates' continuity correction.

**Table 1:** Model selection of the virulence experiment based on Akaike information criteria (AIC). The best fit model estimating morbidity risk of the host (rainbow trout or zebra fish) within time is underlined. *p* value indicates the significance of the term removed from the higher model

Model	AIC	*df*	*p*
H+D+S+NL+H:D+S:D+H:S+NL:H+NL:D+NL:S+H:D:S+NL:H:D+NL:D:S+ H:D:S:NL	557.08	19	
H+D+S+NL+H:D+S:D+H:S+NL:H+NL:D+NL:S+H:D:S+NL:H:D+NL:D:S	555.65	18	.455
H+D+S+NL+H:D+S:D+H:S+NL:H+NL:D+NL:S+NL:H:D+NL:D:S	553.79	17	.705
H+D+S+NL+H:D+S:D+H:S+NL:H+NL:D+NL:S+NL:H:D	550.73	15	.626
H+D+S+NL+H:D+S:D+H:S+NL:H+NL:D+NL:S	554.90	14	.013
H+D+S+NL+H:D+S:D+NL:H+NL:D+NL:S	552.91	13	.946
H+D+S+NL+H:D+S:D+NL:D+NL:S	550.92	12	.924
H+D+S+NL+ H:D+S:D +NL:S	549.17	11	.618
H+D+S+NL+H:D+NL:S	546.50	9	.517
H+D+S+NL+NL:S	544.77	8	.600
H+D+S+NL	545.53	6	.093

H, host; D, dose; S, strain; and NL, nutrient level. + describes the main effects and colon the interactions.

To examine the effect of nutrient level on bacterial replication, the data from the replication study were analyzed using a linear mixed model with bacterial growth (cell count) as a response variable, and nutrient level, bacterial strain (B067 or B398), sampling day (0, 1, 2, 3, 4, 5), and all their possible interactions as explanatory variables. As we intended to compare bacterial cell counts between the days instead of estimating a single time effect, we used factorial sampling day in our analysis. Culture replicate was included in the random effect. The model selection was based on Akaike information criteria and backward stepwise procedure was used to simplify the model (Table 2). When interpreting the effects of the terms in both models, a significance level of 0.05 was used. All statistical analyses were conducted using R software (version 2.15.2) and Lme4 package (R Development Core Team 2011).

**Table 2:** Model selection of the growth experiment based on Akaike information criteria (AIC). The best fit model estimating bacterial count of the strain (B067 or B398) within time is underlined. *p* value indicates the significance of the term if removed from the higher model

Model	AIC	*df*	*p*
S+NL+A+S:NL+S:A+NL:A+S:NL:A+(1 | ID)	3,257.8	26	
S+NL+A+S:NL+S:A+NL:A+(1 | ID)	3,289.0	21	<.001
S+NL+A+S:A+NL:A+(1 | ID)	3,294.4	20	.007
S+NL+A+S:A+(1 | ID)	3,364.5	11	<.001
S+NL+A+ (1 | ID)	3,365.6	10	<.001

S, strain; NL, nutrient level; A, day; and ID, culture replicate (random variable). + describes the main effects, and colon describes the interactions.

## 3 Results

### 3.1 Virulence

**Figure 1:**
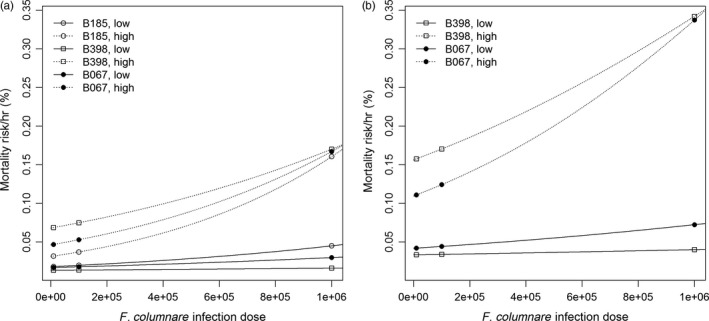
The estimated morbidity risk per hour of (a) zebra fish (*Danio rerio*) and (b) rainbow trout (*Oncorhynchus mykiss*) infected with the high‐virulence strain (B185), low‐virulence strain (B398), or the high‐virulence strain (B067), of *Flavobacterium columnare*. Continuous line indicates low (6.4 ml added growth medium per liter) and dashed line high nutrient level (12.8 ml/L)

The nutrient level, the bacterial dose, and the host species had a significant influence on the fish morbidity risk (Figure 1, Tables 3 and 4), suggesting that *F. columnare* virulence increased in the high‐nutrient environment and with the bacterial density, differing between host species. In more detail, the bacterial strains and doses lead to different infection success in different nutrient concentrations (Figure 1, Table 3), indicating that the effect of the nutrient enrichment on virulence is strain‐specific and depends on bacterial density. Also the increasing effect of bacterial dose on fish morbidity risk differed between the bacterial strains. The rainbow trout had a higher mortality risk than the zebra fish (likely due to its sensitivity to the high temperature used in the experiment), but the response of both hosts to infection was qualitatively similar (Figure 1) (see also Kinnula, Mappes, Valkonen, & Sundberg, 2015). Interestingly in this study, the low‐virulence strain (B398) had the strongest response to nutrient increase as it became virulent in the high‐nutrient treatment (Figure 1). The gill cultivations taken from the infected, moribund hosts were positive for *F. columnare*, whereas the cultivations from the exposed hosts surviving the infection were negative (χ^2^ = 14.1672, *df* = 1, *p *<* *.001).

**Table 3:** The significance and test values of host species, bacterial dose, bacterial strain, and nutrient level on the morbidity risk of the hosts in the virulence experiment. Significant *p* values are denoted in bold

Source	*df*	Deviance	Residual deviance	*p*‐value
Host species	1,124	21.762	398.68	**<.001**
Dose	1,123	46.189	352.49	**<.001**
Strain	2,121	0.913	351.58	.633
Nutrient level	1,120	107.437	244.15	**<.001**
Strain:Nutrient level	2,118	4.576	239.39	.093

**Table 4:** The effect of bacterial dose, nutrient addition, and host species on the morbidity risk of the hosts in the virulence experiment

Source	Estimate	SE
(Intercept)^a^	−3.188	2.787^−1^
Host species (Zebra fish)	−1.018	1.708^−1^
Dose	1.217^−6^	1.598^−7^
Strain B398	−1.448^−1^	2.753^−1^
Strain B067	−3.965^−1^	2.836^−1^
High nutrient level	1.136	2.924^−1^
Strain B398:High nutrient level	2.263^−1^	3.710^−1^
Strain B067:High nutrient level	7.628^−1^	3.751^−1^

a Intercept includes the effects of the host (rainbow trout), the strain (B185), and the low nutrient level.

### 3.2 Bacterial replication in water

**Figure 2:**
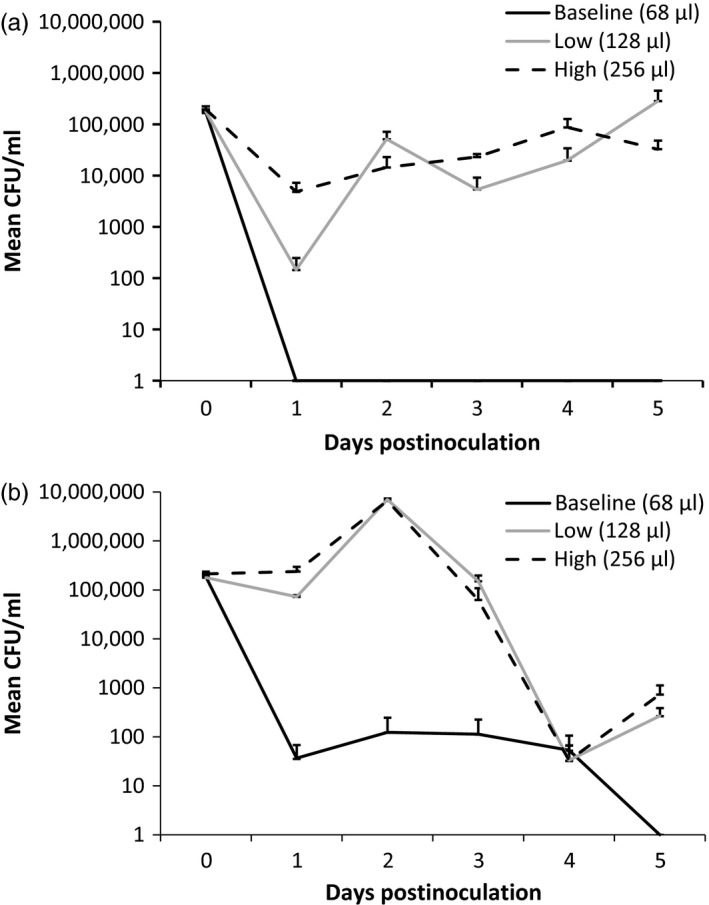
Bacterial densities (mean ± SEM) as colony‐forming units per milliliter of a high‐virulence strain B067 (a) and a low‐virulence strain B398 (b) of *Flavobacterium columnare* at different levels of nutrient enrichment in sterile tap water. The volumes of nutrient medium at each nutrient level (baseline, low, and high) are expressed in the legend. Note the logarithmic scale on y‐axis

The nutrient level and the bacterial strain had a significant effect on the bacterial growth that differed between the sampling days (Figure 2, Tables 5 and 6). We found that the cell count of both the high‐virulence (B067) and the low‐virulence (B398) strains in the presence of nutrient enrichment (low and high nutrient levels) was higher compared to the baseline on days 1, 2, 3, and 5 postinoculation. The replication rate was dependent on the identity of the bacterial strain (Figure 2, Table 5), indicating that the strains responded to the nutrient increase differently. More precisely, the high‐virulence strain appeared to respond rapidly to added nutrients by increased growth and subsequent crash, whereas the low‐virulence strain appeared to respond more slowly, but maintained higher population size for several days (Figure 2).

**Table 5:** The significance and test values of the bacterial strain, the nutrient level, and the day on the bacterial count of the studied strains in the growth experiment. Significant *p* values are denoted in bold

Source	*df*	Sum of squares	Mean square	*F*‐value	*p*‐value
Strain	1	1.637 × 10^13^	1.637 × 10^13^	44.838	**<.001**
Nutrient level	1	7.157 × 10^12^	7.157 × 10^12^	19.610	**.003**
Day	5	7.737 × 10^13^	1.548 × 10^13^	42.400	**<.001**
Strain:Nutrient level	1	6.601 × 10^12^	6.601 × 10^12^	18.086	**.004**
Strain:Day	5	7.988 × 10^13^	1.598 × 10^13^	43.773	**<.001**
Nutrient level:Day	5	3.021 × 10^13^	6.042 × 10^12^	16.554	**<.001**
Strain:Nutrient level:Day	5	3.056 × 10^13^	6.111 × 10^12^	16.745	**<.001**

**Table 6:** The effect of bacterial strain, nutrient level, and sampling day on the bacterial count of the studied strains in the growth experiment

Source	Estimate	Std. Error
(Intercept)^a^	168,306.55	552,754.29
Strain B398	−8,542.41	781,712.61
Nutrient level	160.50	3,254.42
Day 1	−322,466.91	781,712.61
Day 2	−308,855.75	781,712.61
Day 3	−132,965.29	781,712.61
Day 4	−168,293.20	781,712.61
Day 5	−168,389.33	781,712.61
Strain B398:Nutrient level	−25.31	4,602.45
Strain B398:Day 1	161,891.01	1,105,508.58
Strain B398:Day 2	170,618.10	1,105,508.58
Strain B398:Day 3	−36,843.12	1,105,508.58
Strain B398:Day 4	−18,630.99	1,105,508.58
Strain B398:Day 5	116,299.82	1,105,508.58
Nutrient level:Day 1	1,721.90	4,602.45
Nutrient level:Day 2	31,583.62	4,602.45
Nutrient level:Day 3	−198.29	4,602.45
Nutrient level:Day 4	−160.51	4,602.45
Nutrient level:Day 5	−159.22	4,602.45
Strain B398:Nutrient level:Day 1	−1,847.98	6,508.85
Strain B398:Nutrient level:Day 2	−31,726.76	6,508.85
Strain B398:Nutrient level:Day 3	189.52	6,508.85
Strain B398:Nutrient level:Day 4	348.38	6,508.85
Strain B398:Nutrient level:Day 5	−84.91	6,508.85

a Intercept includes the effects of the strain (B067) and inoculation day (day 0).

There was no difference in the growth of the high‐virulence strain between the low and high nutrient levels, but both levels differed from the baseline on days 1, 2, 3, and 5 postinoculation (Figure 2A). However, in line with the findings of the virulence experiment, nutrient addition had a strong influence on the growth of the low‐virulence strain if compared to the baseline (no nutrients added): After population decline on the first day after inoculation, the bacterial cell number stayed continuously high throughout the experiment at low and high nutrient levels, but at baseline, the bacterial population collapsed after inoculation and did no longer replicate (Figure 2b). Bacterial growth differed between sampling days, which probably results from the consecutive growth and decline phases of the bacterial population that are strain‐specific. Also the significant interaction between the nutrient level and the sampling day suggests a connection to the bacterial growth phases, as during the stationary phase, the nutrients are bound to the bacteria and the population can no longer grow, eventually leading to decline of the bacterial population. Therefore, the availability of the nutrients in the system and the number of the living bacterial cells depend on the growth phase of the bacteria and consequently differ between the sampling points.

## 4 Discussion

Ecological factors have been suggested to play a bigger role in disease emergence than evolutionary changes in hosts or their parasites, because the transmission and exposure patterns of pathogens and hosts may be significantly affected by apparently small ecological changes (Schrag & Wiener, 1995). A large amount of organic nutrients is accumulating into the water in aquaculture. This “hypernutrition” has been suggested as an important selective agent facilitating the evolution of pathogenicity. To experimentally test how the outside‐host nutrient level affects virulence of opportunistic pathogens, we exposed rainbow trout (*O. mykiss*) and zebra fish (*D. rerio*) hosts to three strains of *F. columnare* in two different resource concentrations. We chose three different infective doses to investigate the interaction between the nutrient level and the infection dose on the severity of columnaris infection. To identify the mechanisms of action related to *F. columnare* virulence in various nutrient environments, we also studied bacterial replication in sterile dechlorinated tap water with different levels of added nutrients. Our study confirms the significance of increased outside‐host nutrient concentrations for enhanced replication and virulence of opportunists. However, increased bacterial growth could not exhaustively explain the increase in virulence under nutrient‐enriched conditions. The virulence of the studied *F. columnare* strains increased significantly in the high‐nutrient environment compared to the environment with low nutrient addition. In contrast, while nutrient addition also led to improved bacterial replication compared to the baseline level, the growth between the low‐ and high‐nutrient environments did not differ. This result indicates that in addition to the increased outside‐host growth of the pathogen, increased nutrient availability is likely to promote bacterial virulence via virulence factor activation (Penttinen, Kinnula, Lipponen, Bamford, & Sundberg, 2016). Taken together, our results suggest that fluctuations in environmental nutrient conditions can have immediate effects on disease dynamics and outbreak severity. In the long term, increased nutrient levels in aquaculture environment could also act as a potential selective agent for higher pathogen virulence, along with previously acknowledged common culture practices such as high stocking densities and decreased host diversity that decrease the cost of pathogen transmission (Kennedy et al., 2016; Mennerat et al., 2010).

Several experimental studies demonstrate a connection between the pathogen dose and virulence (see e.g., Ebert, Zschokke‐Rohringer, & Carius, 2000; Fellous & Koella, 2010; Regoes, Ebert, & Bonhoeffer, 2002). Consistently, we found the infective dose of *F. columnare* to significantly increase fish morbidity risk, which was also observed in our previous study (Kinnula et al., 2015). This was true for both of the host species, but rainbow trout were found to be more sensitive to columnaris infection than zebra fish. As the hosts still qualitatively responded to doses similarly, this study confirms the suitability of zebra fish as a model in *F. columnare* infection experiments, as suggested in our previous study.

Enhanced availability of nutrients in the water environment has been shown to increase bacterial replication and maintain high bacterial population sizes (Chróst, Adamczewski, Kalinowska, & Skowrońska, 2009; Farjalla, Esteves, Bozelli, & Roland, 2002; Kisand, Tuvikene, & Nõges, 2001; Smith & Schindler, 2009). Furthermore, increased replication rate is often positively correlated with virulence in obligatory microbial pathogens and can thus contribute to the outcome of infection (Brown & Williams, 1985; Read et al., 1999; Smith, 1990). Here, the effect of dose on *F. columnare* virulence was intensified in the high‐nutrient environment, the increase in dose leading to an approximately linear host morbidity risk in the low nutrient concentration and to an exponentially increasing morbidity risk in the high nutrient concentration. Most importantly, the nutrient‐induced virulence increase was more intense for the low‐virulence strain than for the high‐virulence strains, although at the highest bacterial doses, both strains reached similar virulence levels. This additive effect could partly be explained by increased bacterial replication in the higher nutrient concentration and thus a higher infective dose and supports the suggestion that changes in the nutrient environment of a pathogen can lead to rapid changes in virulence (see Ketola et al., 2016). It should, however, be noted that high bacterial dose alone was not sufficient to increase the host mortality risk to similar levels as with high nutrient doses (Figure 1). The high‐ and low‐virulence strains had different growth dynamics in culture, the high‐virulence strain population first growing rapidly and then declining, whereas the low‐virulence strain responded slower but maintained more stable population sizes throughout the experiment. Based on this finding, we suggest that the overlapping growth phases of the bacterial strains co‐occurring in the fish rearing units could promote the maintenance of infective bacterial populations at fish farms, and this effect could be intensified by the high availability of outside‐host nutrients.

There appeared to be no difference in bacterial growth between low‐ and high nutrient concentrations in either of the studied strains, although these same concentrations led to significantly different levels of virulence in the fish. Thus, we propose that in addition to the nutrient‐induced increase in bacterial density, other factors are involved in the higher pathogenicity promoted by the higher nutrient concentration. Bacteria can regulate their virulence factor expression as a response to the environment (Dalebroux, Svensson, Gaynor, & Swanson, 2010; Somerville & Proctor, 2009), and several studies have shown that the increased nutrient availability can prime the expression of virulence‐related genes (Kendall, Day, & Walker, 1913; Berman & Rettger, 1916; Somerville & Proctor, 2009; Torres et al., 2010). A high nutrient concentration has also been found to increase the expression of genes encoding tissue‐degrading enzymes in *F. columnare,* and bacteria grown in high‐nutrient conditions were shown to be more virulent (Penttinen et al., 2016), suggesting that nutrients prime *F. columnare* virulence. Additionally, organic matter and nitrite can also have a positive effect on the adhesion of *F. columnare* to carp (*Cyprinus carpio*) gill tissue (Decostere, Haesebrouck, Turnbull, & Charlier, 1999), although the mechanism is not yet known. Furthermore, processes linked to the nutrient acquisition may facilitate bacterial adaptation to the within‐host environment, which in the end can lead to increases in the pathogenic potential of the bacteria (Le Bouguenec & Schouler, 2011). Therefore, it is possible that the high‐nutrient environment can prime the expression of bacterial virulence factors, resulting in a larger shift in virulence than could be expected purely from the bacterial growth. Monitoring the dynamics of bacterial density, prevalence of the virulent phenotype, and virulence factor expression during infection in different nutrient conditions could provide detailed information on the mechanisms behind this phenomenon.

Currently, it is unclear which one of the medium components could influence *F. columnare* virulence the most, and what kind of effects different commercially used fish feeds have on disease dynamics in rearing units. Here, we used Shieh broth (a common culture media for *F. columnare*) as the source of nutrients in the experimental infections and in the growth experiment. The main ingredient of the broth is peptone (Table S1), a nitrogenous protein derivative, but the medium also contains other nutrients, which are known to act as important growth and virulence factors for microbes, such as phosphorus and iron. Commercial fish feed may contain two orders of magnitude higher nitrogen and phosphorus levels than Shieh broth used in our experiments, and it has been estimated that up to 57% of nitrogen and 76% of phosphorus in fish feed are released back in to the environment (Wang et al., 2013). Nitrogen availability has been linked directly with virulence in fungal and bacterial pathogens (Dalsing, Truchon, Gonzalez‐Orta, Milling, & Allen, 2015; Gouzy et al., 2013; Lee, Morrow, & Fraser, 2013; López‐Berges, Rispail, Prados‐Rosales, & Di Pietro, 2010; Walkowiak & Subramaniam, 2014; Weinhold, Dodman, & Bowman, 1971), including *F. columnare* (Decostere et al., 1997). The role of phosphorus in bacterial virulence is not as clear (but see Frost, Ebert, & Smith, 2008; Romano, Schulz‐Vogt, González, & Bondarev, 2015), although the phosphate (Pho) regulon involved in bacterial phosphate management is known to influence bacterial virulence (Chekabab, Harel, & Dozois, 2014; Lamarche, Wanner, Crépin, & Harel, 2008). In addition, the availability of iron is important for bacterial pathogenesis in several pathogens (e.g., Griffiths, 1991; Rohmer, Hocquet, & Miller, 2011). As a matter of fact, iron has been documented to alter virulence of *F. columnare*, possibly via iron‐scavenging siderophore expression (Guan, Santander, Mellata, Zhang, & Curtiss 2013; Santander, Mellata, Zhang, & Curtiss, 2013; Beck et al., 2015). The presence of these different nutrients and key elements can thus have significant effects on the onset of disease outbreaks by environmentally transmitted pathogens.

To conclude, our results suggest that the presence of excess organic compounds in the aquatic environment can increase bacterial infective dose and consequently lead to a higher risk of infection. Furthermore, high nutrient supply may promote virulence also in avirulent bacterial strains and also increase the risk of disease at low initial bacterial densities. Therefore, maintaining nutrient levels as low as possible in the rearing units can significantly help controlling the onset and progress of disease epidemics in aquaculture.

## Data Archiving Statement

The datasets used in this article are publicly available in Dryad: https://doi.org/10.5061/dryad.57917.
