# Supplementary material for: Higher resource level promotes virulence in an environmentally transmitted bacterial fish pathogen
Source: Evol Appl. 2017 Mar 30;10(5):462–70. doi: 10.1111/eva.12466 (PMC5427672; doi:10.1111/eva.12466)
Supplement: Supplementary file 1 [file EVA-10-462-s001.docx]

**Supplementary Table 1.** The composition of Shieh medium, 1000 ml (according to Decostere et al. 1997).

| Compound | Concentration (g/liter) | Manufacturer |
| --- | --- | --- |
| Peptone | 5.0 | Difco |
| Yeast extract | 0.5 | Difco |
| Sodium acetate | 0.01 |  |
| BaCl_2_(H_2_O)_2_ | 0.01 |  |
| K_2_HPO_4_ | 0.1 |  |
| KH_2_PO_4_ | 0.05 |  |
| MgSO_4_7H_2_O | 0.3 |  |
| CaCl_2_2H_2_O | 0.0067 |  |
| FeSO_4_7H_2_O | 0.001 |  |
| NaHCO_3_ | 0.05 |  |
| Distilled H_2_O (pH 7.2) |  |  |
